# Thermodynamics of π–π Interactions of Benzene and Phenol in Water

**DOI:** 10.3390/ijms23179811

**Published:** 2022-08-29

**Authors:** Dooam Paik, Hankyul Lee, Hyungjun Kim, Jeong-Mo Choi

**Affiliations:** 1 Department of Chemistry, Korea Advanced Institute of Science and Technology, Daejeon 34141, Korea; 2 Department of Chemistry, Pusan National University, Busan 46241, Korea

**Keywords:** pi–pi interaction, intrinsically disordered proteins, QM/MM, solvation effects, enthalpy–entropy compensation

## Abstract

The π–π interaction is a major driving force that stabilizes protein assemblies during protein folding. Recent studies have additionally demonstrated its involvement in the liquid–liquid phase separation (LLPS) of intrinsically disordered proteins (IDPs). As the participating residues in IDPs are exposed to water, π–π interactions for LLPS must be modeled in water, as opposed to the interactions that are often established at the hydrophobic domains of folded proteins. Thus, we investigated the association of free energies of benzene and phenol dimers in water by integrating van der Waals (vdW)-corrected density functional theory (DFT) and DFT in classical explicit solvents (DFT-CES). By comparing the vdW-corrected DFT and DFT-CES results with high-level wavefunction calculations and experimental solvation free energies, respectively, we established the quantitative credibility of these approaches, enabling a reliable prediction of the benzene and phenol dimer association free energies in water. We discovered that solvation influences dimer association free energies, but not significantly when no direct hydrogen-bond-type interaction exists between two monomeric units, which can be explained by the enthalpy–entropy compensation. Our comprehensive computational study of the solvation effect on π–π interactions in water could help us understand the molecular-level driving mechanism underlying the IDP phase behaviors.

## 1. Introduction

The π–π interaction, a typical noncovalent bond involving π-electronic aromatic systems, is one of the prevalent molecular interactions in nature [1]. The association of large biomolecules such as proteins [2,3] and nucleic acids [4] typically involves π–π interaction. Additionally, it plays a significant role in biological processes such as charge transfer [5,6,7], molecular recognition [8,9], and drug delivery [10].

Since Burley identified an interaction between aromatic rings that stabilizes protein structure in 1985 [2], there have been extensive attempts in the realm of quantum chemistry to elucidate the nature of the π–π interaction. The Schlag group obtained benzene dimer interaction energies using Møller–Plesset perturbation theories (MP2 and MP4) and coupled-cluster single-double and perturbative triple (CCSD(T)) theory [11,12,13,14]. They identified three stable geometries of benzene dimer, namely sandwich (S), parallel displaced (PD), and T-shape (T) structures, and discovered that PD and T are the most stable structures with similar binding energies. The Sherrill group further investigated the physical nature and substituent effect of the π–π interaction and provided benchmark-quality potential energy curves for a benzene dimer calculated at the CCSD (T) level with a complete basis set (CBS) limit [15,16,17,18]. These studies provide a fundamental understanding of the π–π interaction and highlight the significance of its role in stabilizing the structure of folded proteins.

Intrinsically disordered proteins (IDPs) are a subclass of proteins lacking cooperatively folded structures in their native state [19], and their biological significance has been extensively studied in recent years. IDPs may undergo liquid–liquid phase separation (LLPS), which is sequence-dependent, and the significance of the π–π interaction in determining their LLPS behavior has been highlighted [20,21]. For example, Gabryelczyk et al. discovered that interactions between tyrosine residues initiate the LLPS of histidine-rich proteins, forming microdroplets [22]. The Pappu group modeled the inter-residue interactions using the stickers-and-spacers framework to predict the phase behavior of IDPs and discovered that the uniform patterning of aromatic residues promotes LLPS and inhibits aggregation [23,24,25]. At present, however, many molecular features of inter-residue interactions, governing the LLPS of IDPs, remain obscure.

While the residues of folded proteins develop the π–π interaction primarily in the hydrophobic pocket, which could be modeled in vacuo, as was likely done in numerous previous quantum chemical studies, the residues of IDPs frequently protrude from the protein backbone toward solvents to develop an interchain π–π interaction. Assuming that the solvation effect’s presence is a major distinction between the inter-residue interactions of folded proteins and IDPs, we explored aromatic π–π interactions in aqueous environments. We examined how water solvation modifies the π–π interaction of benzene and phenol dimers by combining two modern computational chemistry methods: (1) the van der Waals (vdW)-corrected density functional theory (DFT) for an accurate description of direct monomer–monomer interaction energy; (2) DFT in classical explicit solvents (DFT-CES), which is a mean-field quantum mechanics/molecular mechanics (QM/MM) method [26,27,28] enabling an explicit treatment of solvent molecules for a reliable description of solvation free energy. We found that the solvation effect renormalizes the interaction energies between benzene and phenol molecules, the conformational dependence of which can be understood in terms of the changes in the number of hydrogen bonds (HB) before and after dimer formation: water–benzene HB for benzene dimers and water–hydroxyl HB for phenol dimers. The lack of a significant energy difference between in vacuo and in aqua is ascribed to the enthalpy–entropy compensation, which preserves the most stable configuration of a benzene dimer as a T-shape and a phenol dimer as a hydrogen-bonded pair between hydroxyl groups. We expect that our study, which quantifies the interaction strength between aromatic π systems in water, will provide a molecular understanding of the π–π interaction mediated phase behavior of IDPs.

## 2. Methods

### 2.1. Brief Review of DFT-CES/2PT

DFT-CES is a grid-based mean-field QM/MM method that was recently developed by the Kim group [26,27,28]. The mean-field QM/MM method iteratively solves QM optimizations and molecular dynamics (MD) simulations until a self-consistent solution is obtained [29,30,31]. Each QM optimization step relaxes the electron density ($\rho_{\mathrm{QM}}$) and structure ($r_{\mathrm{QM}}$) of the QM particles in the presence of the ensemble-averaged electrostatic potential of the MM component ($\langle V_{\mathrm{MM}}\rangle$), whereas each MD simulation propagates the positions ($r_{\mathrm{MM}}$) and momenta ($p_{\mathrm{MM}}$) of MM particles in the presence of frozen QM particles and $\rho_{\mathrm{QM}}$. Throughout this iterative procedure, known as the self-consistent ensemble-averaged reaction field (SCERF), the free energy functional of the total QM/MM system, $A_{\mathrm{tot}}$, is minimized for the QM nuclear and electronic degrees of freedom [32]:

(1) $$A_{\mathrm{tot}}=E^{\mathrm{KS}}\left[\rho_{\mathrm{QM}};r_{\mathrm{QM}}\right]-k_{B}T\int^{}e^{-\beta H_{\mathrm{MM}}}dr_{\mathrm{MM}}dp_{\mathrm{MM}}$$

Here, the first term indicates the QM subsystem’s internal energy ($E_{\mathrm{QM}}^{\mathrm{int}}$), where $E^{\mathrm{KS}}$ is the Kohn–Sham DFT total-energy functional. The second term represents the Helmholtz free energy of the MM subsystem ($A_{\mathrm{MM}}$), where $H_{\mathrm{MM}}$ is the Hamiltonian regarding the dynamics of MM particles subject to the external potential applied by the frozen QM subsystem (and, thereby, it includes the interaction between the QM and MM regions). $E_{\mathrm{QM}}^{\mathrm{int}}$ can be directly evaluated by DFT calculations, whereas free energy $A_{\mathrm{MM}}$ cannot be directly computed by conventional MD simulations, since such a calculation is often accomplished by turning on and off the constant solute potential [27].

To determine $A_{\mathrm{MM}}$, we utilized the two-phase thermodynamics (2PT) model established by Lin et al. [33], which permits a direct computation of free energy quantities from MD trajectories. The 2PT model separates the total degrees of freedom in a liquid system into gaseous and solid components. The total free energy of the system can then be calculated from the linear combination of the gaseous and solid components, each of which has been studied theoretically using the hard-sphere theory and quasi-harmonic oscillator model, respectively. Notably, the 2PT model has been effective in estimating the thermodynamics and phase behavior of Lennard–Jones particles at various densities [33], the absolute entropy of water molecules [34] and organic solvents [35], and the surface tension of typical liquids [36]. Furthermore, the 2PT model permits the direct decomposition of free energy into enthalpic and entropic components, hence, enhancing the comprehension of the investigated thermodynamic process.

By treating the solutes with QM and the solvents with MM, the DFT-CES/2PT method can be used to evaluate the solvation free energies $\Delta G^{\mathrm{sol}}$. Considering that the solvation process incurs negligible pressure–volume work, $\Delta G^{\mathrm{sol}}$ can be evaluated by computing the $A_{\mathrm{tot}}$. Our previous DFT-CES experiments paired with the 2PT method demonstrated high accuracy in predicting the $\Delta G^{\mathrm{sol}}$ of polar [26] and nonpolar molecules [27] and the thermodynamics and structure of solid–liquid interfaces [37,38,39,40].

### 2.2. Computational Details

We implemented DFT-CES by coupling two open-source programs: the plane-wave (PW) DFT code Quantum Espresso [41] and the large-scale atomic/molecular massively parallel simulator (LAMMPS) [42]. For the DFT calculation, we employed the Perdew–Burke–Ernzerhof (PBE) exchange-correlation functional [43] with Grimme’s D3-type vdW correction [44] and Becke–Johnson damping [45]. The electron–nucleus interaction was described using the projector augmented-wave (PAW) method [46,47] with a 50 Ry kinetic energy cutoff.

For the MD simulation at each SCERF iteration, we performed a canonical ensemble (NVT) MD simulation for 2.5 ns at 300 K using the Nosé–Hoover thermostat [48,49] with a 100-fs damping constant. The final 2 ns of the MD trajectory were used to evaluate the $\langle V_{\mathrm{MM}}\rangle$. The intermolecular interactions of water molecules were represented using a modified TIP3P water potential [50], with bond lengths and angles constrained to equilibrium values using the RATTLE algorithm [51]. Long-range Coulomb interactions were treated using the Ewald summation method [52] with a real-space cut-off of 15 Å.

The simulation box, consisting of one or two QM solute molecules (either benzene or phenol) solvated by 1000 water molecules, has a cubic shape with a box length of 31.2 Å. The vdW pair interaction between QM and MM particles was described using the OPLS-AA force field [53], and SCERF iterations were conducted until the difference in $E_{\mathrm{QM}}^{\mathrm{int}}$ was less than 0.1 kcal/mol. DFT structural optimization may place the QM system too close to the site of averaged MM particles because the DFT-CES involves Pauli repulsion between the QM and MM regions only in the MD component utilizing the pairwise potential, but not in the DFT part. To prevent such a technical issue with phenol systems, the H atom from the hydroxyl group in phenol was maintained at its original position in the structure optimized in vacuo, while no constraints were imposed on the benzene system.

**Figure 1:**
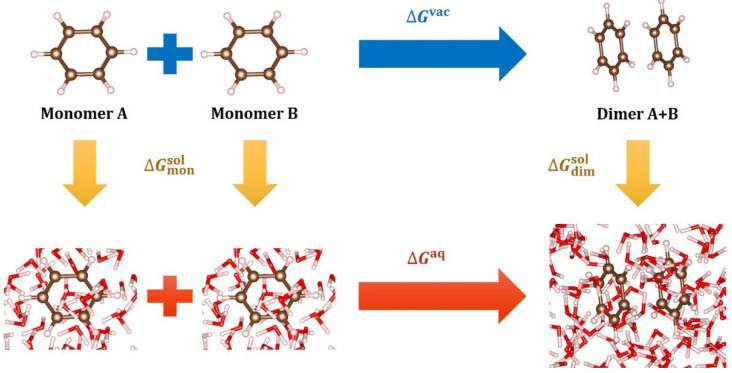
Thermodynamic cycle describing aromatic π–π interaction in aqueous solution. The free energy of association in water ($\Delta G^{\mathrm{aq}}$) can be calculated using the free energy of association in vacuum ($\Delta G^{\mathrm{vac}}$) and the solvation free energy difference ($\Delta \Delta G^{\mathrm{sol}}$) between a dimer ($\Delta G_{\dim}^{\mathrm{sol}}$) and two monomers ($\Delta G_{\mathrm{mon}}^{\mathrm{sol}}$). Different colors indicate different atom types: white—hydrogen; brown—carbon; and red—oxygen (same for the following figures).

We then calculated the free energy of association in water, $\Delta G^{\mathrm{aq}}$, using the thermodynamic cycle shown in Figure 1. The free energy of association in vacuo, $\Delta G^{\mathrm{vac}}$, was calculated using vdW-corrected DFT energetics, wherein the vibrational free energies, including the zero-point energy (ZPE) contribution, were corrected using harmonic oscillator partition functions. We estimated the vibrational frequencies for computational efficiency using the Gaussian orbital code, Jaguar version 10.8 [54], at the level of PBE-D3/AUG-cc-pVTZ-PP(-F). Then, using separate DFT-CES/2PT calculations, the solvation free energies of the monomers and their dimer complexes, i.e., $\Delta G_{\mathrm{mon}}^{\mathrm{sol}}$ and $\Delta G_{\dim}^{\mathrm{sol}}$, were calculated, leading to $\Delta G^{\mathrm{aq}}=\Delta G^{\mathrm{vac}}+\;\Delta \Delta G^{\mathrm{sol}}$, where $\Delta \Delta G^{\mathrm{sol}}=\Delta G_{\dim}^{\mathrm{sol}}-2\Delta G_{\mathrm{mon}}^{\mathrm{sol}}$.

### 2.3. Exploration of Different Conformations

**Figure 2:**
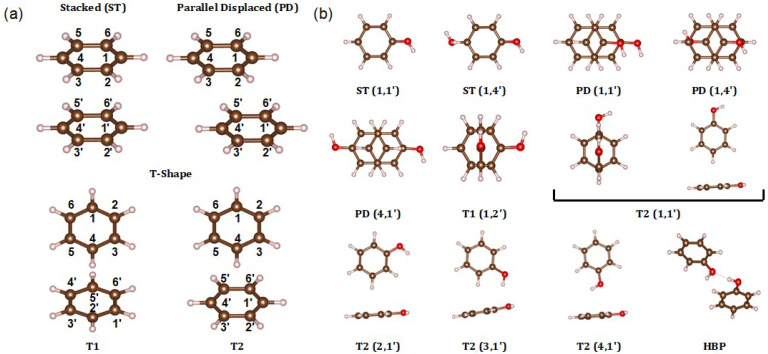
(**a**) Geometric conformations of benzene dimer. Each carbon from the upper (1~6) and lower (1′~6′) benzene is assigned a number. (**b**) Geometric conformations of phenol dimer. Each structure is designated by the position of the ring component (ST, PD, T1, T2, or HBP) and the number of substituted carbons with a hydroxyl group from the upper (1~6) and lower (1′~6′) phenol.

Herein, we focused on the π–π interactions in benzene and phenol dimers. As in earlier quantum chemical investigations [11,12,13,14,15,16,17,18], stacked (ST), parallel-displaced (PD), and T-shape (T) conformations were explored for benzene dimers (Figure 2a). It is to be noted that two T conformations with different symmetries (labeled as T1 and T2) were investigated for completeness.

Phenol dimers can assume an extensive variety of different conformations depending on the relative locations of the hydroxyl (OH) groups attached to the phenyl rings. To systematically investigate the impact of the relative locations of OH groups, we first constructed two distinct ST conformations of the phenol dimer: one by positioning OH groups with the closest distance, and the other by positioning OH groups with the farthest distance, which are labeled ST (1,1′) and ST (1,4′), respectively (see Figure 2b). By horizontally shifting one of the phenyl rings of ST (1,1′) and ST (1,4′), we constructed three distinct PD conformations of phenol dimers, labeled PD (1,1′), PD (4,1′), and PD (1,4′), as shown in Figure 2b.

For the T conformations, the OH group location in the horizontally laid phenyl ring (whose carbons are labeled using primed numbers in Figure 2a) is thought to be less important than the OH group location in the vertically standing phenyl ring (whose carbons are labeled using unprimed numbers in Figure 2a). Indeed, we discovered virtually no difference in $\Delta G^{\mathrm{vac}}$ with different OH group locations in the horizontally laid phenyl ring (Figure S1). Therefore, we chose two representative cases, T1 (1,2′) and T2 (1,1′), as shown in Figure 2b. To explore the effect of the OH group location on the vertically standing phenyl ring, we further chose the T2 (2,1′), T2 (3,1′), and T2 (4,1′) conformations.

Finally, as a hydrogen-bonded pair (HBP) can develop between the hydroxyl groups of two phenol molecules, the corresponding geometry was also added for further investigation, although it cannot be strictly classified as a π–π interaction.

## 3. Results and Discussion

### 3.1. Benchmark: Dimer Binding Energy in Vacuo & Monomer Solvation Energy

Table 1 shows the binding energies of the benzene and phenol dimers in vacuo ($\Delta E^{\mathrm{vac}}$), which were calculated using the difference in the DFT self-consistent field (SCF) energies between the dimer and monomer, $\Delta E^{\mathrm{vac}}=E_{\mathrm{dimer}}^{\mathrm{SCF}}-2E_{\mathrm{monomer}}^{\mathrm{SCF}}$. Incorporating the entropic corrections and the ZPE, the calculation of the corresponding free energy, $\Delta G^{\mathrm{vac}}$, is allowed as listed in Table 1. To evaluate the accuracy of PBE-D3, we compared the $\Delta E^{\mathrm{vac}}$ values to the results of previous studies using high-level wavefunction theory, such as coupled cluster theory. For the S, PD, and T1 conformations of the benzene dimer, the results from the PBE-D3/PW level and the estimated CCSD(T)/aug-cc-pVQZ* level agreed within approximately 0.2 kcal/mol [18]. When available, we further confirmed that the $\Delta E^{\mathrm{vac}}$ values of phenol dimers correlate well with the MP2 and coupled-cluster level data from previous studies [55,56].

To reliably predict $\Delta G^{\mathrm{aq}},$ the solvation free energy, $\Delta G^{\mathrm{sol}}$, must be accurately calculated using the thermodynamic cycle depicted in Figure 1. In this regard, we compared the estimated and observed values of $\Delta G^{\mathrm{sol}}$ for the benzene and phenol monomers. Several implicit solvation methods implemented in the Jaguar suite [54], including the Poisson–Boltzmann finite element method (PBF) [57]; polarizable continuum model (PCM) [58]; and Minnesota solvation models (SM6 and SM8) [59,60] were also evaluated. The results are summarized in Table 2, and we found that the DFT-CES values agree with the experimental values [61], in stark contrast with the implicit solvation methods, which predict $\Delta G^{\mathrm{sol}}$ with gross errors. It is clear that the existence of the OH group hugely determines the solvation behavior in water, accounting for significant differences of $\Delta G^{\mathrm{sol}}$ between benzene and phenol, and this behavior can be extrapolated to polyphenols such as quercetin [62].

### 3.2. Free Energy of Association in Aqueous Environment

**Figure 3:**
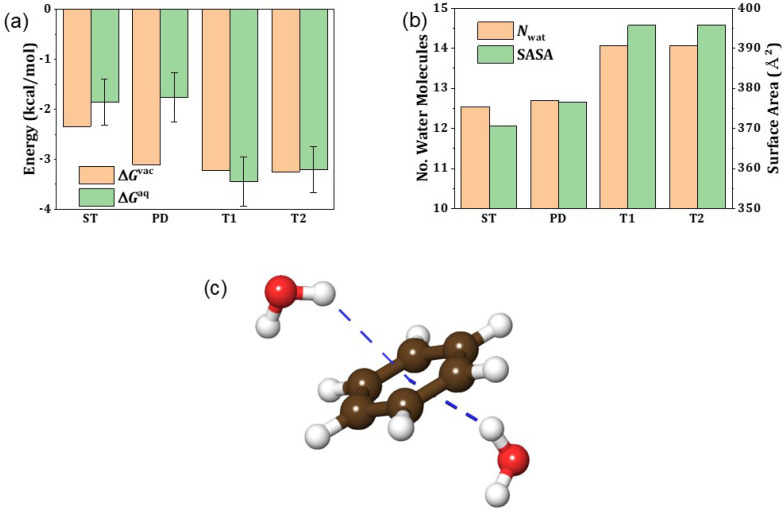
(**a**) Association free energy of benzene dimers in vacuum ($\Delta G^{\mathrm{vac}}$) and in solution ($\Delta G^{\mathrm{aq}}$). (**b**) Average number of water molecules in the first solvent shell ($N_{\mathrm{wat}}$) and solvent accessible surface area (SASA) corresponding to the benzene dimer association in water. (**c**) Schematic illustrating the benzene–water hydrogen bonds.

Based on the successful descriptions of $\Delta E^{\mathrm{vac}}$ and $\Delta G^{\mathrm{sol}}$ enabled by PBE-D3 and DFT-CES, respectively, we investigated the $\Delta G^{\mathrm{aq}}$ of a benzene dimer for the ST, PD, T1, and T2 conformations (Figure 3a). We observed that the solvation effect slightly weakens the benzene dimer interaction for ST and PD conformations by 0.5–1 kcal/mol, while it remains almost unchanged for the T1 and T2 conformations. This alters the most favorable conformation of the benzene dimer; the PD and T conformations are almost equally stable in vacuum, but the T conformations preferentially attain stability in water.

Intriguingly, the solvent-accessible surface areas (SASAs) of the two T conformations were greater than those of the other two conformations, resulting in more water molecules surrounding the solute molecules (Figure 3b). In contrast, hydrophobic interactions occur between nonpolar and hydrophobic molecules, such as benzene, which tend to aggregate in water while minimizing their solvent-exposed surface. This is because the aromatic ring of benzene is not simply nonpolar and can develop two benzene–water hydrogen bonds on both sides of the phenyl ring (Figure 3c) [63]. As the association into T conformations only breaks one benzene–water hydrogen bond, while that into ST or PD conformations breaks two hydrogen bonds, the benzene dimer association in water favors the T conformations. Indeed, our earlier research indicated that breaking the benzene-water hydrogen bond requires approximately 1 kcal/mol [64], which nearly corresponds to the current difference in $\Delta G^{\mathrm{sol}}$ between the PD and T conformations.

**Figure 4:**
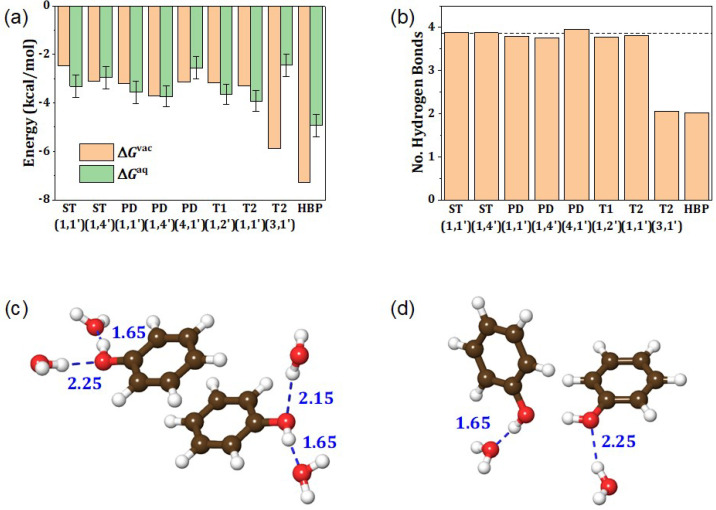
(**a**) Association free energy of phenol dimers in vacuum ($\Delta G^{\mathrm{vac}}$) and in water ($\Delta G^{\mathrm{aq}}$). (**b**) Average number of water–hydroxyl hydrogen bonds present in phenol dimers. The dashed line represents twice the average number of hydrogen bonds of phenol monomer. (**c**,**d**) Schematic illustrating the water–hydroxyl hydrogen bonds of (**c**) PD (4,1′) and (**d**) HBP conformation. The numbers are H-bond distances in angstroms, which are taken from the first peak locations of radial distribution functions shown in Figure S4.

Figure 4a shows the $\Delta G^{\mathrm{aq}}$ values of phenol dimers in various conformations. Note that the T2 (2,1′) and T2 (4,1′) conformations were excluded from further discussion because they were discovered to be extremely unstable in water, resulting in either a structure largely deviated from its original conformation or a positive value of $\Delta G^{\mathrm{aq}}$ (Figure S2). Except for the T2 (3,1′) and HBP conformations, we found smaller differences among different conformations and hydroxyl group positions. Moreover, $\Delta G^{\mathrm{aq}}$ values were comparable to $\Delta G^{\mathrm{vac}}$ values within a 1 kcal/mol margin. However, for the T2 (3,1′) and HBP conformations, the solvation effect significantly weakened the dimer association.

The major reason for the strongest binding in vacuum of the T2 (3,1′) and HBP conformations can be found in the direct interaction between the two OH groups. The HBP conformation creates an intermolecular HB between the two phenols. The T2 (3,1′) conformation also causes the OH group at the vertically upright phenyl ring to point toward the O atom of the OH group at the horizontally placed phenyl ring (see Figure 2), forming a weak HB. Because OH groups are solvated by water molecules via strong hydrogen bonds in the aqueous phase, when the association process develops a direct interaction between two OH groups (e.g., for the case of T2 (3,1′) and HBP), the loss of water–hydroxyl hydrogen bonds is inevitable. Figure 4b depicts the variation in the number of water–hydroxyl HB during the phenol dimer association. HB connectivity is shown in Figure S3. As the OH groups in the ST and PD conformations protrude toward the water phase, they can form ~2 HBs with water molecules for each OH group, as depicted in Figure 4c for the PD (4,1′) case. On the other hand, the OH groups in the T2 (3,1′) and HBP conformations mutually interact in the buried space, and only one solute–solvent hydrogen bond per OH group is permitted, as illustrated in Figure 4d. This is ascribed to the weakened interaction between T2 (3,1′) and HBP phenol dimers in water, whereas $\Delta G^{\mathrm{aq}}$ and $\Delta G^{\mathrm{vac}}$ are comparable for the other conformations. Nevertheless, despite the loss of water–hydroxyl hydrogen bonds, the HBP phenol dimer remains the most stable phenol dimer conformation in water.

We compared the values of $\Delta G^{\mathrm{vac}}$ and $\Delta G^{\mathrm{aq}}$ with the ring-to-ring distances between the dimers of benzene and phenol (Figure S5). Both $\Delta G^{\mathrm{vac}}$ and $\Delta G^{\mathrm{aq}}$ have negative relationships with the distances, indicating that the most stable configurations (i.e., T-shape for benzene dimer and HBP for phenol dimer) have the longest R-R distances. We also investigated the electrostatic potential surfaces of their configurations (Figure S6) and conclude that the ring components of benzene or phenol tend to become apart from each other to avoid the overlapping of charge densities, hence, developing strong π–π interactions.

### 3.3. Enthalpy–Entropy Compensation in Solvation Effect

**Figure 5:**
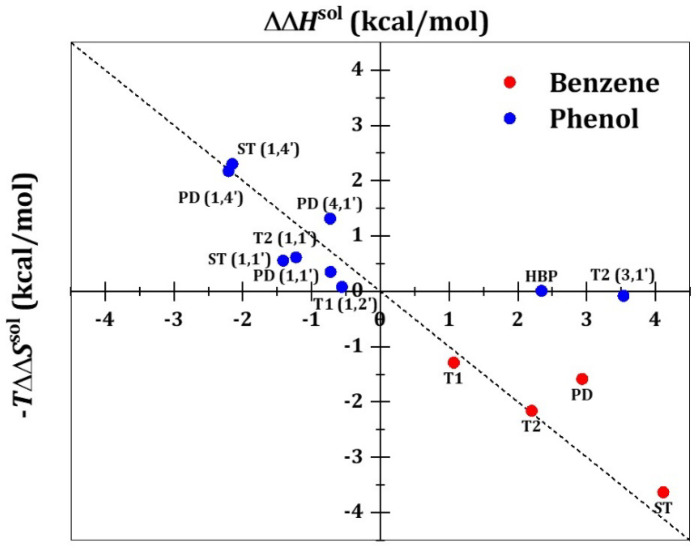
Enthalpic ($\Delta \Delta H^{\mathrm{sol}}$) and entropic contributions ($-T\Delta \Delta S^{\mathrm{sol}}$) of the solvation free energy difference ($\Delta \Delta G^{\mathrm{sol}}$) between a dimer and two monomers. The dashed line represents y=−x.

Despite being non-negligible, the association free energy difference in vacuo and in aqua is mostly limited within 1.4 kcal/mol for all benzene and phenol dimer cases, except for the T2 (3,1′) and HBP phenol dimers, which have solute–solute hydrogen bonds (as also supported by the natural bond orbital analyses shown in Figure S7). As the association free energy difference is determined by the $\Delta \Delta G^{\mathrm{sol}}$, as illustrated in the thermodynamic cycle (Figure 1), we decomposed $\Delta \Delta G^{\mathrm{sol}}$ into the enthalpic contribution, $\Delta \Delta H^{\mathrm{sol}}$, and the entropic contribution, $-T\Delta \Delta S^{\mathrm{sol}}$, which are plotted in Figure 5.

Except for the two outliers, T2 (3,1′) and HBP phenol dimers, the enthalpy–entropy compensation behavior in $\Delta \Delta G^{\mathrm{sol}}$ was well-defined. Moreover, as the corresponding $\Delta \Delta H^{\mathrm{sol}}$ and $-T\Delta \Delta S^{\mathrm{sol}}$ values are distributed around the *y* = −*x* line, we discovered that the solvation effects of enthalpy and entropy cancel each other out at 300 K, yielding small differences between $\Delta G^{\mathrm{aq}}$ and $\Delta G^{\mathrm{vac}}$ within 1.4 kcal/mol. Interestingly, both $\Delta \Delta H^{\mathrm{sol}}$ and $\Delta \Delta S^{\mathrm{sol}}$ were negative for phenol dimers (except for T2 (3,1′) and HBP conformations), while $\Delta \Delta H^{\mathrm{sol}}$ and $\Delta \Delta S^{\mathrm{sol}}$ were positive for benzene dimers. Thus, it is inferred that enthalpy drives the coalescence of two hydration shells of phenol monomers into one, whereas entropy drives the fusion of benzene hydration shells. Consequently, the former can be classified as a nonclassical hydrophobic association, whereas the latter can be considered as an example of classical association.

During the dimerization of phenol into the T2 (3,1′) or HBP conformation, the enthalpy disfavors the coalescence of two hydration shells ($\Delta \Delta H^{\mathrm{sol}}>0$) owing to the loss of the hydroxyl–water hydrogen bond, but the entropy is insufficient to compensate ($\Delta \Delta S^{\mathrm{sol}}$~0). This causes the solvation thermodynamics of the T2 (3,1′) and HBP conformations to deviate from the trend established by the enthalpy–entropy compensation, resulting in a significantly weakened association free energy in water. However, despite this energy cost in water, the HBP conformation of phenol dimers has the strongest $\Delta G^{\mathrm{aq}}$ among all considered conformations; it is also stronger than the $\Delta G^{\mathrm{aq}}$ of the benzene dimer.

### 3.4. Relationships with Phase Behaviors of IDPs

Benzene and phenol are analogs of phenylalanine (Phe) and tyrosine (Tyr) side chains, respectively. Even with a small number of charged residues, a subset of IDPs containing a significant number of Phe/Tyr residues can drive liquid–liquid phase separation. One well-known example is the N-terminal low-complexity domain of FUS (fused in sarcoma), which contains 24 Tyr residues and few charged residues (2 Asp) [65].

The strongest benzene dimer interaction is −3.44 kcal/mol for the T1 conformation, and the strongest phenol dimer interaction is −4.93 kcal/mol for the HBP conformation; both are negative and sufficiently large, so they can promote the association, but the phenol dimer has a stronger binding. Other theoretical investigations [66,67] have demonstrated that the interaction strength of the associating units is positively correlated with the LLPS propensity. Indeed, previous experiments [23] have demonstrated that Phe and Tyr can replace each other without significantly altering the phase behaviors, but Tyr induces a stronger tendency toward LLPS than Phe [68,69].

## 4. Conclusions

In this study, the thermodynamics of benzene and phenol dimer association were investigated by determining the free energy of π–π interactions in water. Using vdW-corrected DFT and DFT-CES methods, we quantitatively estimated the free energy of dimer association in water, $\Delta G^{\mathrm{aq}}$. Each component term (free energy of dimer association in vacuum and solvation free energy) was sufficiently accurate to be compared with available high-level coupled cluster-level calculations and experimental values. Interestingly, we discovered that the solvation effect only marginally modifies the π–π interaction free energies, unless a direct hydrogen-bond-like interaction develops between the two monomers. This trend can be explained in terms of enthalpy–entropy compensation, which clarifies that entropy and enthalpy drive the association of benzene and phenol dimers, respectively. We expect that this study will yield quantitatively reliable free-energetic values for π–π interactions in water and provide a fundamental understanding of the molecular driving force for the liquid–liquid phase separation of IDPs.

## Figures and Tables

**Table 1:** Estimated binding energies ($\Delta E^{\mathrm{vac}}$ ) and binding free energies ($\Delta G^{\mathrm{vac}}$ ) of benzene and phenol dimer in vacuum. The listed values are in kcal/mol. $\Delta E^{\mathrm{vac}}$ from other studies were calculated using ^(a)^ estimated CCSD(T)/aug-cc-pVQZ*, which is a modified aug-cc-pVQZ basis set lacking g functions on carbon and f functions on hydrogen [18]. ^(b)^ MP2/M062X/6-311++G(d,*p*) [55]. ^(c)^ CCSD(T)/CBS [56].

		Present Study		Others
		$\Delta G^{\mathrm{vac}}$	$\Delta E^{\mathrm{vac}}$	$\Delta E^{\mathrm{vac}}$
**Benzene** **Dimer**	ST	−2.34	−1.70	−1.70 ^(a)^
	PD	−3.11	−2.43	−2.63 ^(a)^
	T1	−3.22	−2.66	−2.61 ^(a)^
	T2	−3.25	−2.66	
**Phenol** **Dimer**	ST (1,1′)	−2.46	−1.69	−1.61 ^(b)^
	ST (1,4′)	−3.11	−2.42	−2.76 ^(b)^
	PD (1,1′)	−3.19	−2.45	−3.62 ^(b)^
	PD (1,4′)	−3.71	−3.00	
	PD (4,1′)	−3.14	−2.47	−4.26 ^(b)^
	T1 (1,1′)	−3.12	−2.57	
	T1 (1,2′)	−3.18	−2.64	
	T1 (1,3′)	−3.34	−2.83	
	T2 (1,1′)	−3.31	−2.79	
	T2 (1,2′)	−3.10	−2.56	
	T2 (1,3′)	−3.26	−2.75	
	T2 (1,4′)	−3.28	−2.77	
	T2 (2,1′)	−3.50	−2.99	
	T2 (3,1′)	−5.90	−5.66	
	T2 (4,1′)	−3.65	−3.12	
	HBP	−7.29	−6.87	−6.81 ^(c)^

**Table 2:** Calculated solvation free energies ($\Delta G^{\mathrm{sol}}$) of benzene and phenol monomer using PBF, PCM, SM6, SM8, and DFT-CES method. The listed values are in kcal/mol. The experimental values of $\Delta G^{\mathrm{sol}}$ are from the hydration dataset of a previous study about solvated binding free energies [61].

	Benzene	Phenol
PBF	−0.09	−6.72
PCM	−2.52	−5.45
SM6	−3.27	−5.60
SM8	−3.61	−5.63
DFT-CES	−0.85	−6.72
Experiment	−0.87	−6.62

## Data Availability

Not applicable.

## Supplementary Materials

The following supporting information can be downloaded at: https://www.mdpi.com/article/10.3390/ijms23179811/s1.
