# Supplementary material for: Thermodynamics of π–π Interactions of Benzene and Phenol in Water
Source: Int J Mol Sci. 2022 Aug 29;23(17):9811. doi: 10.3390/ijms23179811 (PMC9456204; doi:10.3390/ijms23179811)
Supplement: Supplementary file 1 [file ijms-23-09811-s001.zip › ijms-1857115-supplementary.pdf]

Supplementary Material

Thermodynamics of π-π Interactions of Benzene and Phenol in Water

Dooam Paik[1†], Hankyul Lee[1†], Hyungjun Kim[1]* and Jeong-Mo Choi[2]*

Affiliations:

[1]Department of Chemistry, Korea Advanced Institute of Science and Technology, Daejeon 34141, Republic of Korea.

[2]Department of Chemistry, Pusan National University, Busan 46241, Republic of Korea.

*Corresponding authors: linus16@kaist.ac.kr (H.K.) and jmchoi@pusan.ac.kr (J.-M.C.)

†These authors contributed equally to this work.

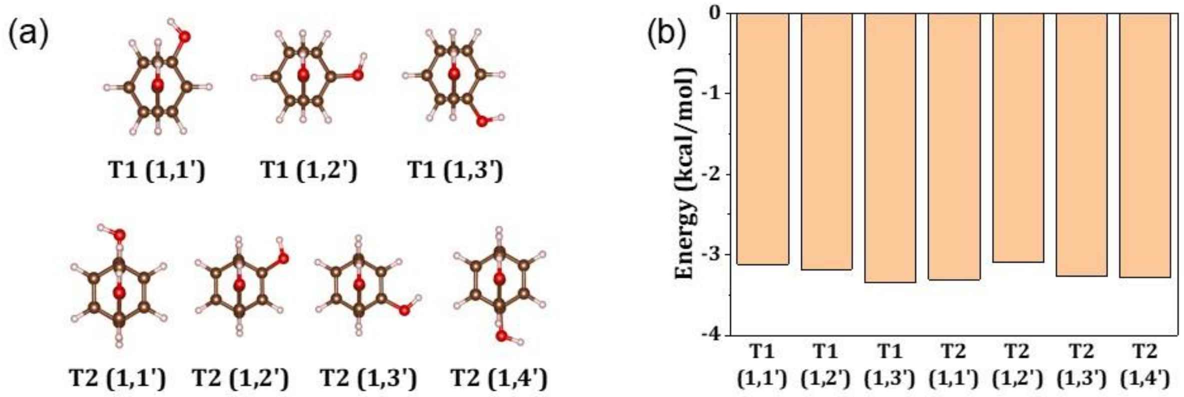

Figure S1. (a) Different conformations of phenol dimers with varying the location of OH group in the horizontally laid phenyl ring. The numbers in parenthesis represent the location of hydroxyl groups. (b) Association free energy of phenol dimers calculated in vacuum (ΔG^{vac}).

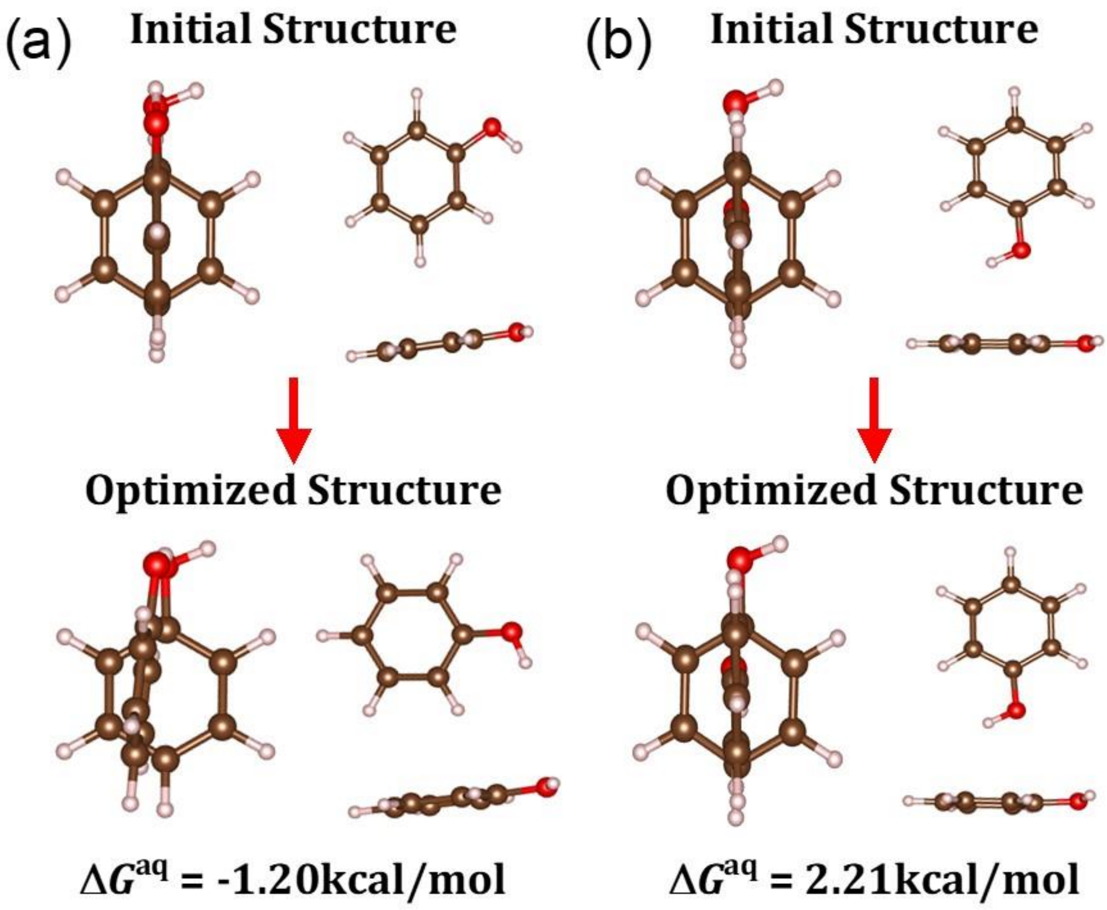

Figure S2. Before and after geometry optimizations of (a) T2(2,1') and (b) T2(4,1') conformations in water. T2(2,1') conformation is too unstable to maintain the original conformation, and T2(4,1') shows a positive binding free energy in water, that is ΔG^{aq} = 2.21 kcal/mol, disfavoring the dimer association.

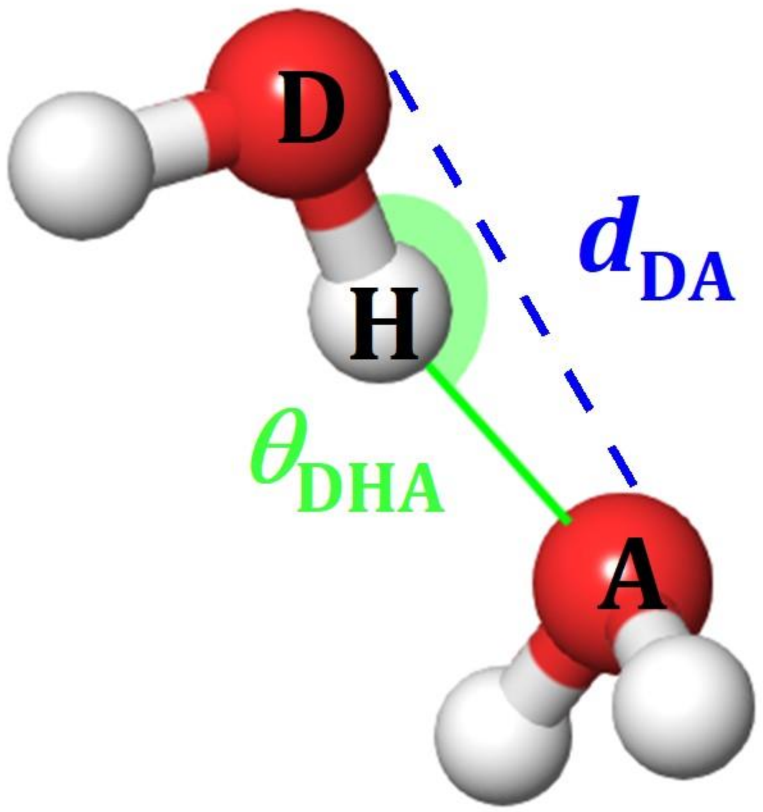

Figure S3. The hydrogen bond is defined when the distance between the donor and acceptor oxygen atoms (d_{DA}) is shorter than 3Å and the angle $\angle O_A H O_D$ is larger than 120º.

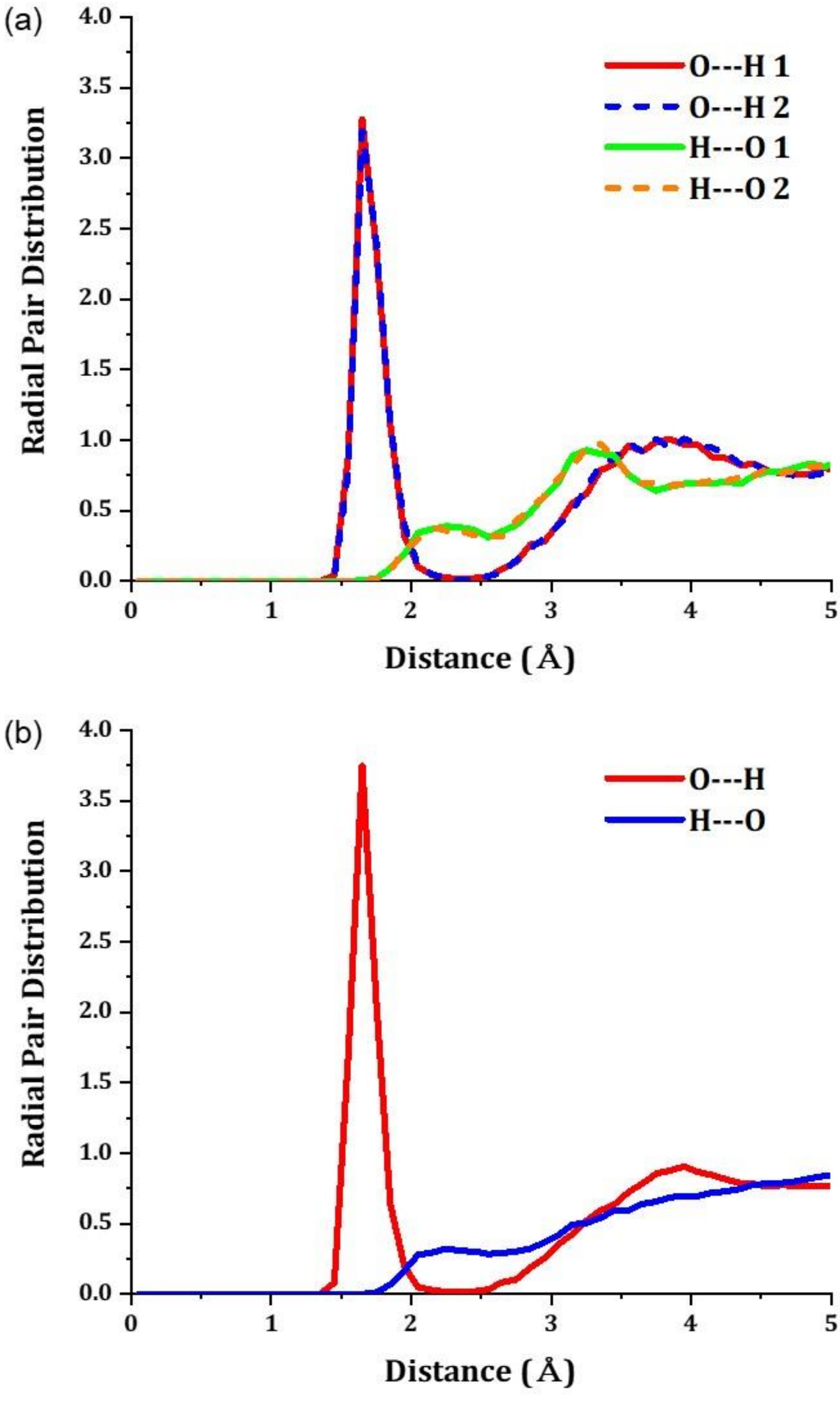

Figure S4. Radial pair distributions for (a) PD(4,1') and (b) HBP conformations of phenol dimer; O---H is for the pair of O-atom of the water and H-atom of the hydroxyl group, and H---O is for the pair of H-atom of the water and O-atom of the hydroxyl group.

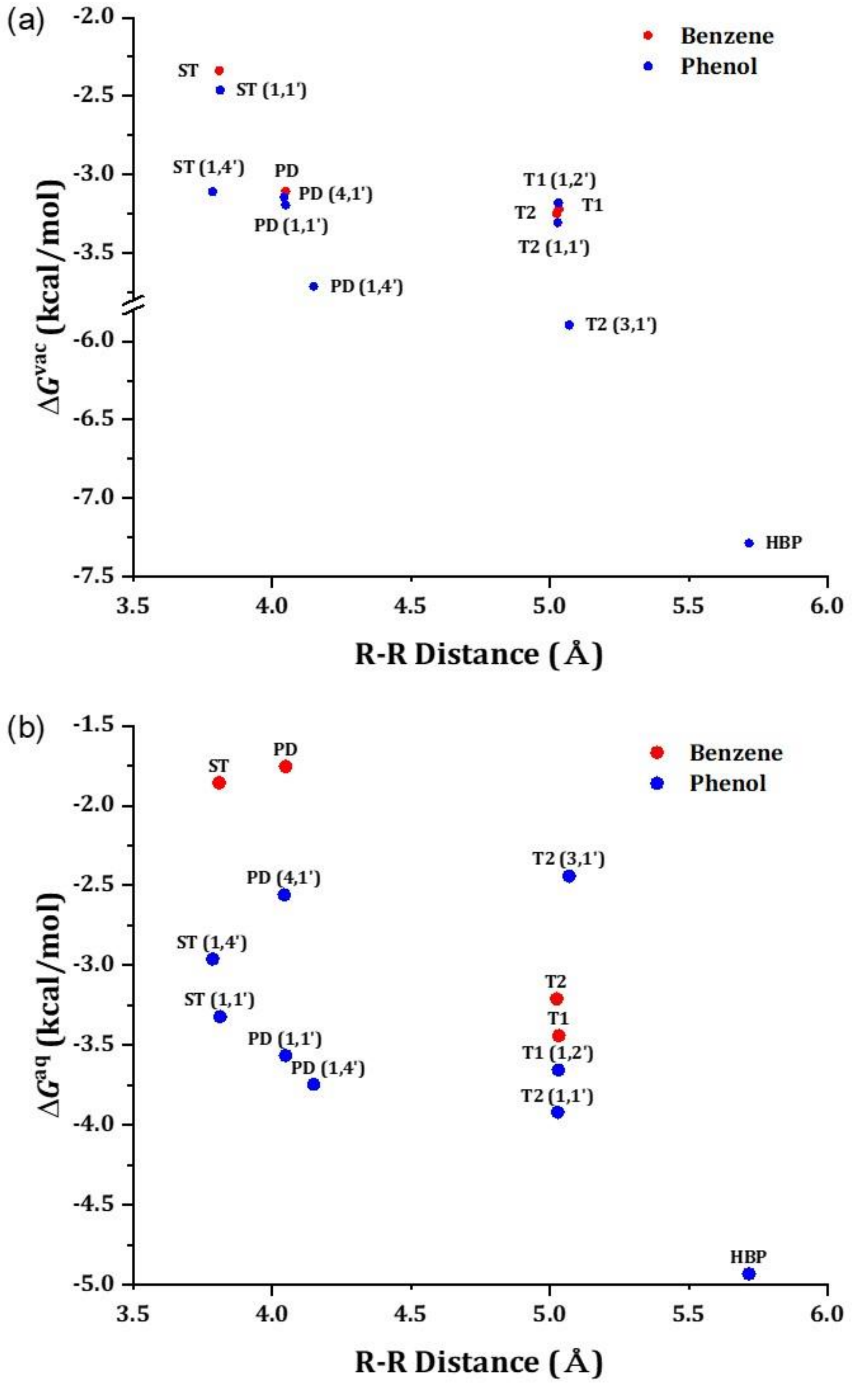

Figure S5. Relationship between ring-to-ring (R-R) distance and association free energies (a) in vacuum (ΔG^{vac}) and (b) in water (ΔG^{aq}).

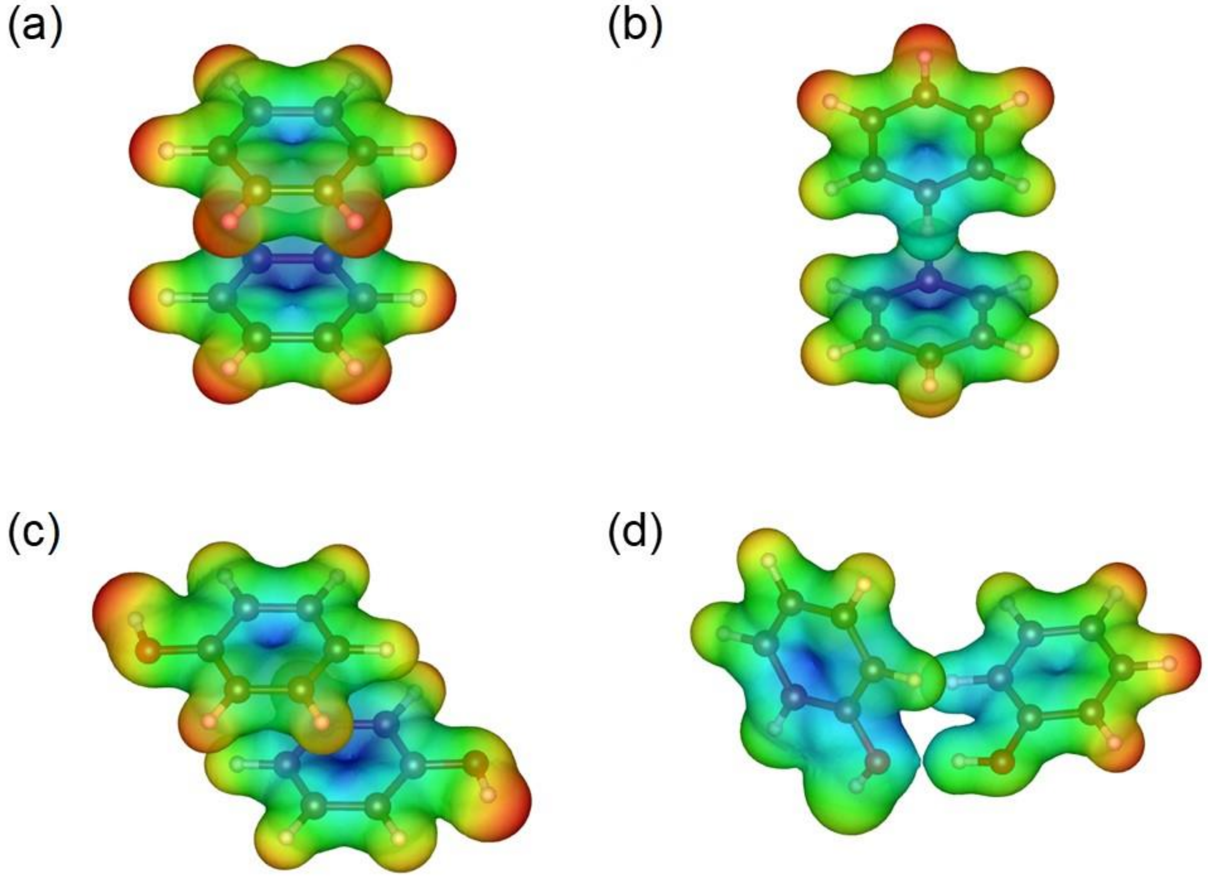

Figure S6. Electrostatic potential surfaces of (a) ST, (b) T1 conformations of benzene dimer, and (c) PD(4,1’), (d) HBP conformations of phenol dimer.

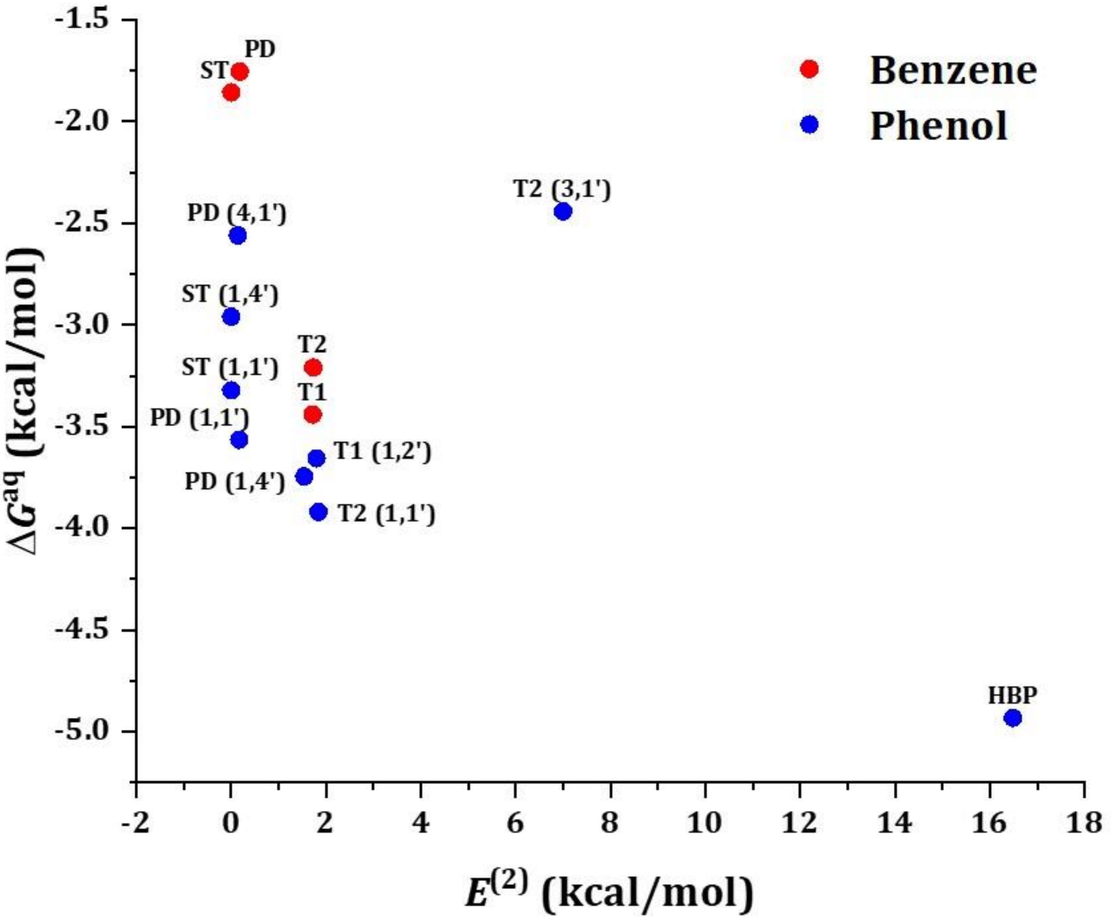

Figure S7. Relationship between the solute-solute interaction energies ($E^{(2)}$) and association free energies in water (ΔG^{aq}). $E^{(2)}$ was calculated using the second-order perturbation theory analysis of Fock matrix in natural bond orbital (NBO) basis.
